# Mannose and Hyaluronic Acid Dual-Modified Iron Oxide Enhances Neoantigen-Based Peptide Vaccine Therapy by Polarizing Tumor-Associated Macrophages

**DOI:** 10.3390/cancers14205107

**Published:** 2022-10-18

**Authors:** Ying Nie, Lu Shi, Yanan Zhang, Yunfei Guo, Hongchen Gu

**Affiliations:** NanoBiomedical Research Center, School of Biomedical Engineering & Med-X Research Institute, Shanghai Jiao Tong University, 1954 Huashan Road, Shanghai 200030, China

**Keywords:** macrophage polarization, iron oxide, mannose, hyaluronic acid, tumor-associated macrophages, cancer vaccine

## Abstract

**Simple Summary:**

Cancer vaccine therapy is promising, though its efficacy is compromised in an immunosuppressive tumor microenvironment. Tumor-associated macrophages (TAMs) have the potential to be repolarized to an antitumor subtype, and their antigen-presenting ability might enhance the efficacy of cancer vaccination. Here, we aimed to develop a nanoparticle with adjuvant effect that could be a carrier for neoantigen-based vaccine to target and repolarize TAMs in situ. Therefore, we prepared a hyaluronic acid and mannose dual-modified iron oxide nanoparticle, and the enhanced efficiency of nanoparticle intake of macrophages was confirmed. It could repolarize macrophages and outperformed a commercialized iron oxide nanoparticle, ferumoxytol. Combined with peptides, this nanoparticle strongly inhibited TC1 tumor growth, and 40% of mice reached complete regression. This is the first report using mannose, hyaluronic acid, and iron oxide to target TAMs and achieve ideal outcomes in vivo. This study provides a facile nanoplatform for neoantigen-based vaccine therapy and a reference for repolarizing TAMs to promote immunotherapy.

**Abstract:**

Neoantigen-based cancer vaccine therapy is a breakthrough in the field of immunotherapy. However, it is difficult for vaccines against neoantigens to overcome the immunosuppressive microenvironment, where tumor-associated macrophages (TAMs) play a significant role. Herein, we report an iron oxide nanoparticle modified with hyaluronic acid and mannose to reshape the tumor microenvironment by targeting and repolarizing TAMs from protumor M2 to antitumor M1 phenotype. Mannose decoration could confer the nanoparticle-enhanced TAM targeting ability, while hyaluronic acid and iron oxide could repolarize M2-like macrophages both in vitro and in vivo. Combined with antigenic peptides, this nanovaccine could significantly increase the infiltration of CD8^+^ T cells into tumor tissue and strongly activate dendritic cells in sentinel lymph nodes. Finally, we used the dual-modified nanoparticles to first convert the tumor microenvironment and then the nanovaccine administration in a TC1 tumor model to further enhance efficacy. This strategy inhibited tumor growth and achieved a 40% cure rate in mice (two of five). In summary, this study provides a potent and rationally designed nanoadjuvant to enhance antitumor efficiency and facilitate delivery of neoantigen vaccines by repolarizing TAMs and harmonizing immune cells.

## 1. Introduction

Neoantigen-based cancer vaccines are a promising avenue for cancer therapy [1,2,3,4]. Personalized vaccines predicated on neoantigens can elicit tumor-specific T cell responses and avoid broad-spectrum toxicity caused by traditional cancer therapies. Benefiting from next-generation sequencing, detecting tumor-specific mutations in individual patients is certainly feasible. However, there are some limitations that hinder better efficacy of cancer vaccines, one of which is the insufficiency in overcoming immunosuppression in tumor microenvironment (TME) [4,5,6].

In TME, macrophages (TAMs) are abundant and highly plastic. Recent research has shown that TAMs play an important role in the immunosuppression of the tumor environment and generally show tumor-supportive phenotypes (i.e., M2-like subtype) [7,8,9,10]. The depletion or targeting inhibition of the recruitment of TAMs indeed benefits some cancer patients, thereby facilitating the development of some anticancer drugs, such as anti-colony stimulating factor 1 receptor, anti-chemokine (C-C motif) ligand 2, anti-signal regulatory protein α, and anti-cluster of differentiation 40 [11,12,13]. However, toxicity should be taken into account because of the broad distribution of macrophages in the whole body. In addition, macrophages are antigen-presenting cells (APCs) and they express costimulatory signals to activate T lymphocytes once they are skewed to M1 phenotype [14,15]. Thus, repolarizing TAMs in situ is a promising approach to empower efficacy of cancer vaccine.

Iron oxide nanoparticles have been found to have the ability to repolarize macrophages from M2 to antitumor M1 phenotype [16]. Daldrup-Link’s group successfully inhibited the development of liver metastases in mice following pretreatment with the FDA-approved iron oxide, ferumoxytol [17]. Subsequent studies further confirmed the effectiveness of iron oxide in repolarizing macrophages and partially achieving tumor inhibition [18,19]. However, bare iron oxide alone induces a limited immune response and cannot completely inhibit tumor growth.

Polysaccharide molecules have been discovered to stimulate innate immune cells like macrophages because they usually represent pathogen invasion. In 2008, researchers demonstrated that low-molecular-weight hyaluronic acid activated the self-defense of the epithelium [20]. In 2015, Gemeinhart et al. reported that low-molecular-weight hyaluronic acid preferentially activated proinflammatory pathways in macrophages [21]. Thus, the modification of hyaluronic acid (HA) to iron oxide nanoparticles might further enhance macrophage repolarization. Mannose is known to target M2 macrophages, whose mannose receptors (CD206) are one typical marker for M2 phenotype identification [12,22,23].

**Scheme 1:**
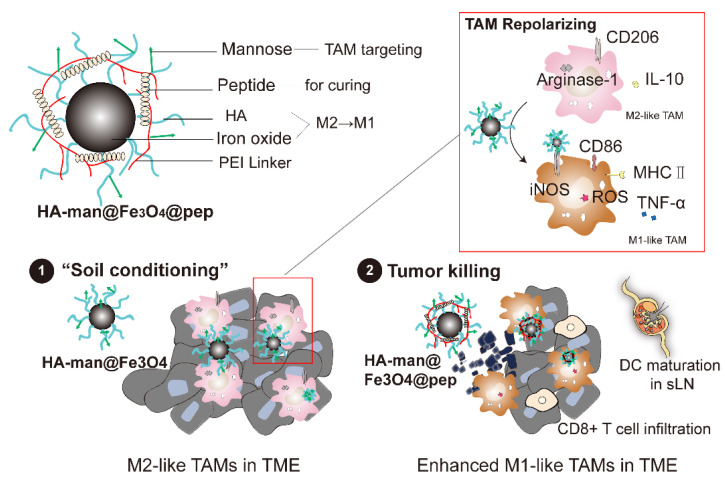
The components of rationally designed nanoparticles and the nanostrategy of combination therapy. This strategy consists of two steps, as follows: (1) tumor-associated macrophage polarization by HA-man@Fe_3_O_4_ to improve “immune soil” in TME; (2) tumor killing conveyed by nanocomplex vaccination.

Therefore, combination therapy with HA and mannose dual-modified iron oxide and neoantigen vaccines may be an effective approach to actively target M2-like TAMs, empower repolarization and promote efficacy of neoantigen-based vaccines. We hypothesized that the conversion of TME with iron oxide and HA would first alleviate immunosuppression to improve the “soil” for immune system and then promote the effectiveness of neoantigen vaccines (Scheme 1). Commercialized iron oxide ferumoxytol was set in comparison because they have been proven to have a repolarization effect and thus antitumor efficacy as aforementioned. Remarkably, our modified nanoparticles outperformed ferumoxytol both in vitro and in vivo and two of five tumor-bearing mice reached complete regression with the two-step treatment. This nanoparticle-based delivery system provides an efficient and generalizable adjuvant strategy to targeting and repolarizing TAMs, which might overcome immunosuppression and achieve enhanced efficacy of neoantigen-based vaccine.

## 2. Materials and Methods

### 2.1. Preparation of Hyaluronic Acid-Coated Fe_3_O_4_ Nanoparticles (HA@Fe_3_O_4_) and Oxidized Hyaluronic Acid-Coated Iron Oxide Nanoparticles (HA@Fe_3_O_4_-O_2_)

HA@Fe_3_O_4_ was synthesized using the classic chemical coprecipitation method [24]. First, we obtained oxygen-free water by boiling ultrapure water for 5 min and maintaining it in a nitrogen environment. Sodium hyaluronate (1.7 g) was dissolved in 15 mL of oxygen-free water, and 0.6 mL hydrochloric acid for protonation. Subsequently, 2 g of FeCl_3_·6H_2_O and 1.4 g of FeSO_4_·7H_2_O in 5 mL of oxygen-free water were added. The solution was placed in a three-necked flask and stirred at 85 °C for 10 min under nitrogen atmosphere. Twenty milliliters of ammonia (7.5 wt.%) was added to the flask at a rate of one drop per second. The mixture was stirred at 85 °C for 30 min and then cooled. The black product was centrifuged at 11,000× *g* for 30 min. The upper layer was placed in a dialysis bag (6000–8000 Da) and dialyzed for 48 h to remove the free molecules. The product was concentrated via rotary evaporation to obtain a stable HA@Fe_3_O_4_ dispersion. We obtained oxidized hyaluronic acid-coated iron oxide nanoparticles (HA@Fe_3_O_4_-O_2_) by blowing air into HA@Fe_3_O_4_ at 70 °C for 6 h.

### 2.2. Synthesis of Hyaluronic Acid and Mannose Co-Modified Fe_3_O_4_ Nanoparticles (HA-man@Fe_3_O_4_)

HA-man@Fe_3_O_4_ was synthesized by conjugating HA@Fe_3_O_4_ with d-mannosamine hydrochloride. Briefly, HA@Fe_3_O_4_ was dispersed in 10 mм of 2-(N-morpholino) ethanesulfonic acid (MES)-T solution and mixed with 43.1 mg of D-mannosamine hydrochloride. The dispersion was stirred at 37 °C for 30 min to achieve adsorption saturation. Subsequently, we added 19.15 mg of 1-ethyl-3-(3-dimethylaminopropyl) carbodiimide hydrochloride (EDC) to the dispersion. The mixture was stirred at 37 °C for 3 h. Then, the mixture was filtered three times using an ultrafiltration centrifuge tube (50,000 Da cutoff). The product was dispersed in ultrapure water to obtain a stable HA-man@Fe_3_O_4_ dispersion. Experiments concerning different feeding ratio is detailed in Table 1.

### 2.3. Synthesis of Mannose-Modified Polyethyleneimine (PEI-man)

First, we dispersed 230.5 mg of D-polymannuronic acid sodium salt in 30 mL of 10 mM MES-T buffer. Subsequently, we dissolved 222.8 mg of EDC and 401.27 mg of N-hydroxysuccinimide (NHS) in 10 mL of 10 mм MES-T buffer, and added it to a D-polymannuronic acid sodium salt solution under stirring. After stirring for 30 min at room temperature, 100 mg of polyethyleneimine (branched MW = 10,000 Da) was dissolved in 30 mL of 10 mм MES-T buffer and mixed with the solution. We stirred the mixture at room temperature for 24 h. Eventually, the mixture was placed in a dialysis bag (6000–8000 Da) and dialyzed for 48 h. The mixture was lyophilized to obtain the yellow spongy final product, modified polyethyleneimine (PEI-man). We performed Fourier-transform infrared spectroscopy (Nicolet 6700, Thermo Fisher, Waltham, MA, USA) to confirm the synthesis.

### 2.4. Synthesis of Nanovaccine Consisting of Neoantigen Peptides and HA-man@Fe_3_O_4_ (HA-man@Fe_3_O_4_@pep)

Ovalbumin (OVA) and neoantigen peptides were synthesized by GenScript Biotechnology Co., Ltd. (Nanjing, China). Table 2 summarizes the amino acid sequences. HA-man@Fe_3_O_4_ particles were suspended in ultrapure water and mixed with neoantigen peptides. The mass ratio of the particles in Fe to the peptides was 10:1. We dissolved PEI-man in ultrapure water and added it to the mixture. Subsequently, the pH of the mixture was adjusted to 5 with 1 M HCl solution under ultrasonic dispersion. The hydration particle size and zeta potential of HA-man@Fe_3_O_4_@pep were characterized and transmission electron microscopy (TEM) images were obtained. All samples were diluted with ultrapure water to an optimal concentration before sending to the Instrumental Analysis Center in Shanghai Jiao Tong University for TEM imaging.

To characterize the load capacity, we applied a series of mass ratios (50:1, 25:1, 10:1, 5:1, and 1:1) of HA-man@Fe3O4 in Fe and OVA. Following this, the supernatants were collected after ultrafiltration. The remaining OVA in the supernatant was measured using a bicinchoninic acid protein assay kit.

### 2.5. Characterization of Synthesized Nanoparticles

We obtained transmission electron microscopy (TEM; Talos F200X G2, Thermo Fisher, USA) images to reveal the morphology of the NPs. We measured the hydration particle sizes and zeta potentials using a dynamic light scattering spectrometer (NanoZS, Malvern, UK). Fourier-transform infrared spectroscopy (Nicolet 6700, Thermo Fisher, USA) and thermogravimetric analysis (TGA 8000, PerkinElmer, Norwalk, CT, USA) were performed to analyze the surface-coating molecules. The X-ray diffractions of the nanoparticles were characterized following freeze-drying (D8 Advance Davinci, Bruker, Fremont, CA, USA).

### 2.6. Cellular Uptake

RAW 264.7 cells were provided by the Stem Cell Bank, Chinese Academy of Sciences (Shanghai, China), and cultured in complete Dulbecco’s modified Eagle’s medium (10% fetal bovine serum and 2% penicillin–streptomycin solution) at 37 °C under 5% CO_2_ atmosphere. The cells were pretreated with 20 ng mL^−1^ of mouse recombinant interleukin (IL)-4 for 24 h. We added HA@Fe_3_O_4_, HA@Fe_3_O_4_-O_2_, HA-man@Fe_3_O_4_, and ferumoxytol (Feraheme^®^, AMAG Pharmaceuticals, Waltham, DE, USA) to the medium at 200 μg mL^−1^ in Fe. An equal volume of phosphate-buffered saline (PBS) was added to the control group. Following 24 h, the cell samples were washed three times in PBS and incubated with 100 μL of 50% hydrochloric acid solution in a 96-well plate at 37 °C for 15 min. Then, 100 μL 5% potassium ferrocyanide solution was added to each well at 37 °C for 30 min. The concentrations of the iron standard curve were set at 100 ppm, 50 ppm, 25 ppm, 12.5 ppm, and 6.25 ppm, respectively, and treated as above. We measured the absorbance at 650 nm using a microplate reader. For microscopic imaging, the cells were incubated with 10% potassium ferrocyanide solution for 10 min, followed by 10% potassium ferrocyanide solution containing 10% HCl for an additional 30 min. We used nuclear fast red to visualize the cell nuclei.

### 2.7. Effect of RAW 264.7 Cells on Repolarization In Vitro

Briefly, RAW 264.7 cells were pretreated with 20 ng mL^−1^ of mouse recombinant IL-4 for 24 h. These cells were cocultured with HA@Fe_3_O_4_, HA@Fe_3_O_4_-O_2_, HA-man@Fe_3_O_4_, and ferumoxytol at 200 μg mL^−1^ in Fe. We added an equal volume of PBS to the control group. The culture medium was collected for enzyme-linked immunoassay (ELISA) analysis. We used mouse tumor necrosis factor (TNF)-α and IL-10 ELISA kits (Thermo Fisher, USA), according to the manufacturer’s instructions. The cells were collected for flow cytometry. First, they were washed with PBS and incubated with TruStain fcX™ (anti-mouse CD16/32) for 20 min on ice. Cells were then incubated with PE/Cyanine7 anti-mouse/human CD11b (BioLegend, San Diego, CA, USA) and Brilliant Violet 421™ anti-mouse CD86 (BioLegend, USA) for 20 min on ice, according to the manufacturer’s suggestions. Subsequently, they were incubated with fixation buffer (BioLegend) and intracellular staining buffer (BioLegend) for 10 min each. Then, they were incubated with APCanti-mouse CD206 (BioLegend, USA) for 20 min on ice. We used a BD FACSAria III flow cytometer (BD Biosciences, Franklin Lake, NJ, USA) for the analysis.

### 2.8. Reactive Oxygen Species (ROS) Assay

RAW 264.7 cells were pretreated with 20 ng mL^−1^ mouse recombinant IL-4 for 24 h, followed by HA@Fe_3_O_4_, HA@Fe_3_O_4_-O_2_, HA-man@Fe_3_O_4_, and ferumoxytol at 200 μg mL^−1^ in Fe. The cells were collected and analyzed using a reactive oxygen species (ROS) detection assay kit (Biovision, San Francisco, CA, USA, K936) according to the manufacturer’s instructions. We used a microplate reader to measure the fluorescence intensity (Ex/Em = 495/529 nm). A fluorescence microscope was used to obtain cell images.

### 2.9. Quantitative RT-PCR (qRT-PCR) Analysis of the Repolarization of RAW 264.7 Cells

Specifically, RAW 264.7 cells were pretreated with 20 ng mL^−1^ of mouse recombinant IL-4 for 24 h and cultured with HA@Fe_3_O_4_, HA@Fe_3_O_4_-O_2_, HA-man@Fe_3_O_4_, and ferumoxytol at 200 μg mL^−1^ in Fe. We extracted the total RNA of these samples with TRIzol™ Plus RNA Purification kit (Invitrogen, Carlsbad, CA, USA) based on the manufacturer’s instructions. We analyzed the RNA samples using the HiScript II One Step quantitative reverse transcription polymerase chain reaction (qRT-PCR) SYBR Green kit (Vazyme, Nanjing, China). Table S1 outlines the sequences of RT-qPCR primers.

### 2.10. TC1 Inhibition by Repolarized RAW 264.7 In Vitro

TC1 (ATCC CRL-2493) cells were cultured in 24-well plates at a density of 2 × 10^5^ cells per well. RAW 264.7 cells were cultured in an upper 0.4 μm transwell chamber at a density of 2 × 10^4^ cells per well. The E/T ratio was therefore 1:10, and the transwell prevented macrophage migration. We added ferumoxytol, HA@Fe_3_O_4_, HA@Fe_3_O_4_-O_2_, HA-man@Fe_3_O_4_, and HA-man@Fe_3_O_4_@pep to the Transwell to achieve a final Fe concentration of 200 μg mL^−1^. After 24 h, TC1 cells were collected and analyzed using an Annexin V-FITC/PI Apoptosis Detection Kit (Yeasen, Shanghai, China).

### 2.11. Tumor Growth Inhibition

We purchased C57BL/6 mice (female, 3–5 weeks old) from Lingchang Biotech, 2012 (Shanghai, China). All animal experiments were approved by the Animal Welfare Ethics Committee of Shanghai Jiaotong University. Specifically, 2 × 10^5^ of TC1 cells suspended in 100 μL of PBS was subcutaneously injected into the right flank of the mice. The mice were randomized into six groups and intratumorally injected with PBS, peptides (2 mg kg^−1^), HA-man@Fe_3_O_4_ (20 mg kg^−1^ in Fe), HA-man@Fe_3_O_4_ + peptides (20 mg kg^−1^ in Fe, 2 mg kg^−1^ in peptide), ferumoxytol + peptides (20 mg kg^−1^ in Fe, 2 mg kg^−1^ in peptide), or HA-man@Fe_3_O_4_ + HA-man@Fe_3_O_4_@pep (20 mg kg^−1^ in Fe, 2 mg kg^−1^ in peptide) upon the tumor volume reaching 100 mm^3^. Tumor volumes were recorded every alternate day and they were calculated with formula V = (tumor length) × (tumor width)^2^ × 0.5. The study design was approved by the Animal Welfare Ethics Committee of Shanghai Jiaotong University.

### 2.12. Flow Cytometry Analysis of Tumor Tissue and Sentinel Lymph Nodes

We collected the tumor tissue and sentinel lymph nodes posttreatment and treated them with collagenase A for 1.5 h at 37 °C. The lysates were washed with PBS and filtered through a 70 μm nylon mesh. The cells were incubated with the Zombie Red Fixable Viability Kit for 10 min to exclude the dead cells. Then, they were treated with TruStain fcX™ (anti-mouse CD16/32) on ice and incubated with the following antibodies for flow cytometry: APC/Fire 750 anti-mouse CD45, PE/Cyanine7 anti-mouse/human CD11b, Brilliant Violet 510 anti-mouse F4/80, APC anti-mouse CD206, and Brilliant Violet 421 anti-mouse CD86 markers to analyze the tumor-associated macrophages. T cells were analyzed using APC/Fire™ 750 anti-mouse CD45, APC anti-mouse CD3ε, FITC anti-mouse CD4, PerCP/Cy5.5 anti-mouse CD8a, and PE anti-mouse Foxp3 markers. APC/Fire™ 750 anti-mouse CD45, PE anti-mouse CD11c, PE/Cyanine7 anti-mouse/human CD11b, PerCP/Cy5.5 anti-mouse MHCII, and Brilliant Violet 421™ anti-mouse CD86 markers were used to analyze the dendritic cells (DCs).

### 2.13. Quantitative RT-PCR (qRT-PCR) Analysis of Tumor Tissue

Tumor tissue was collected following treatment and extracted with TRIzol™ Plus RNA Purification kit (Invitrogen, USA) according to the manufacturer’s instructions. RNA samples were analyzed using the HiScript II One Step qRT-PCR SYBR Green kit (Vazyme, China). Table S1 gives the sequences of RT-qPCR primers.

### 2.14. Statistical Analysis

All statistical analyses were based on multiple independent experiments repeated at least twice. Statistical analyses were performed using SPSS and one-way analysis of variance (ANOVA), followed by Student’s *t*-test. Statistical differences were defined as significant for * *p* < 0.05 and highly significant for ** *p* < 0.01 and *** *p* < 0.001.

## 3. Results

### 3.1. Synthesis and Characterization of HA@Fe_3_O_4_, HA@Fe_3_O_4_-O_2_ and HA-man@Fe_3_O_4_

The synthesis method was modified from the one-pot preparation reported by Soleymani [25]. We first coated iron oxide with HA and the nanoparticles in this step were abbreviated as HA@Fe_3_O_4_. Then HA@Fe_3_O_4_ were modified with mannose to obtain our dual-modified nanoadjuvant (HA-man@Fe_3_O_4_). We also developed oxidized HA@Fe_3_O_4_ (HA@Fe_3_O_4_-O_2_) to be in comparison in the following experiments to examine the effects of oxidization process.

**Figure 1:**
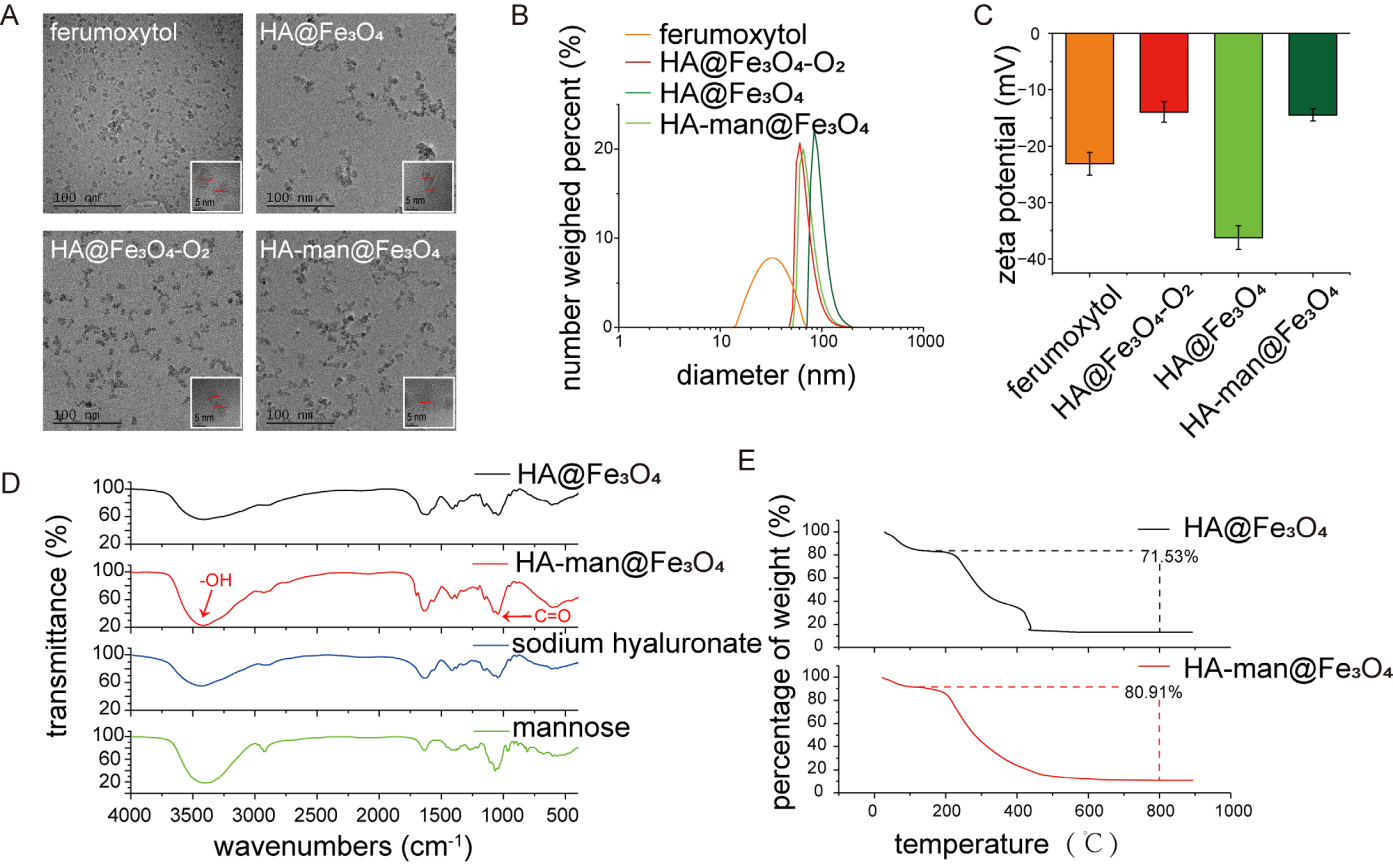
Characterization of iron oxides. (**A**) Transmission electron microscope (TEM) images of ferumoxytol, HA@Fe_3_O_4_, HA@Fe_3_O_4_-O_2_, and HA-man@Fe_3_O_4_; (**B**,**C**) size distribution and zeta potentials of ferumoxytol, HA@Fe_3_O_4_, HA@Fe_3_O_4_-O_2_ and HA-man@Fe_3_O_4_; (**D**) infrared spectra of HA@Fe_3_O_4_, HA-man@Fe_3_O_4_, sodium hyaluronate and mannose; and (**E**) thermogravimetric analysis (TGA) curves of HA@Fe_3_O_4_ and HA-man@Fe_3_O_4_. Error bars indicate at least three independent experiments.

The core size of the HA@Fe_3_O_4_ particles was similar to that of ferumoxytol, as displayed in the TEM images (Figure 1A). Six to nine nanometers was the most common core size. Following the oxidation process, the core size and hydraulic diameter of HA@Fe_3_O_4_-O_2_ remained unchanged (Figure 1A,B); nonetheless, the crystal conformation of HA@Fe_3_O_4_-O_2_ was different from the Fe_3_O_4_ conformation of HA@Fe_3_O_4_ (Figure S1). The crystal conformation of HA@Fe_3_O_4_-O_2_ was not typical of α-Fe_2_O_3_ ether, which might be attributable to the incomplete oxidation process.

Following mannose modification, the HA-man@Fe_3_O_4_ particles clustered together, resulting from decreased surface charges (Figure 1A,C). This phenomenon might explain the enlarged hydraulic diameter of HA-man@Fe_3_O_4_ (Figure 1B). Figure 1C depicts the zeta potential values of the particles. All particles displayed a negative zeta potential, similar to that of ferumoxytol. The zeta potential of HA-man@Fe_3_O_4_ was considerably smaller than that of HA@Fe_3_O_4_, which could be explained by the consumption of the carboxyl groups during mannose modification and indicated successful modification. We measured the zeta potential values of HA-man@Fe_3_O_4_ with a series of mannose modification efficiencies to confirm this hypothesis (Table 1).

**Table 1:** The effect of different feeding ratios on the modified mannose amount of HA-man@Fe_3_O_4_.

Molar Ratio ^(a)^	Concentration of Filtrate Containing Mannose [mg]			AVE	STD	Amount of Remaining [mg]	Amount of Feeding [mg]	Load Efficiency ^(b)^ [%]	Modification Efficiency ^(c)^ [%]
5:1	21.52	22.96	23.85	22.78	0.96	88.94	107.75	17.45	87.27
2:1	6.85	6.65	6.48	6.66	0.15	26.01	43.10	39.66	79.32
1:1	4.44	4.36	4.26	4.35	0.07	17.00	21.55	21.11	21.11
1:2	2.66	2.54	2.48	2.56	0.07	10.00	10.78	7.27	3.63
1:5 ^(d)^	2.41	2.38	2.36	2.38	0.02	9.31	4.31	-	-
1:10 ^(d)^	1.81	1.76	1.71	1.76	0.04	6.87	2.15	-	-

^(a)^ The molar ratio is the ratio of the number of moles of amino groups in mannose to the number of moles of carboxyl groups on the surface of HA@Fe_3_O_4_. ^(b)^ The load efficiency calculation method is (amount of feeding − amount of remaining)/amount of feeding. ^(c)^ Modification efficiency is calculated as the molar amount of modified mannose/the molar amount of carboxyl groups on the surface of HA@Fe_3_O_4_. ^(d)^ The feeding mannose concentration is lower than the detection limit of the detection method, and the amount of remaining obtained by the fitting calculation is higher than the initial amount of feeding, which is of no practical significance.

We used infrared spectroscopy to examine whether the coating and modification were successful. As shown in Figure 1D, HA coating of HA@Fe_3_O_4_ was confirmed by transmittance peak at 3300 cm^−1^ which indicated the introduction of hydroxyl. The infrared spectroscopy of HA-man@Fe_3_O_4_ displayed enhanced transmittance at 1045 cm^−1^, which indicated the introduction of carbonyl of mannose. We also found that HA accounted for 71.53% of the total weight of the particles and that mannose accounted for approximately 10% of the total weight of the particles (Figure 1D,E).

### 3.2. Enhanced Targeting towards M2-like Macrophages

**Figure 2:**
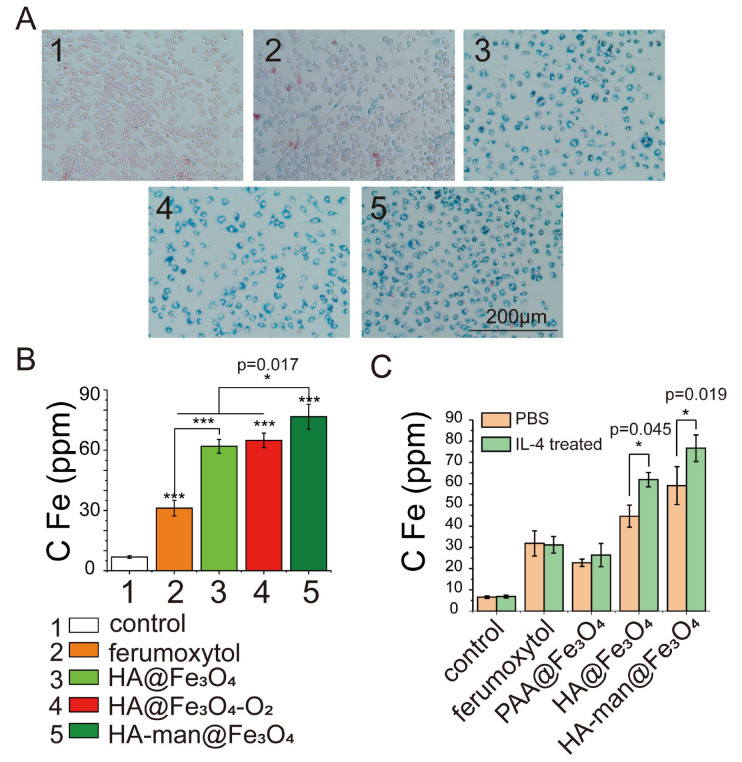
RAW264.7 cells cocultured with different iron oxides in vitro. (**A**) Prussian blue staining images of RAW 264.7 cells; (**B**) Fe amount in RAW 264.7 cells. Cells were incubated with 200 μg mL^−1^ iron oxides in Fe for 24 h. (**C**) The targeting abilities of iron oxide nanoparticles with different coating molecules towards macrophages. PBS or IL-4 stimulated RAW264.7 cells were cocultured with materials for 24 h before testing. Error bars indicate at least three independent experiments. *p*-value has been calculated by the one-way ANOVA test. * *p* < 0.05 and *** *p* < 0.001.

Mannose has the potential to target macrophages via mannose receptor-mediated internalization [26]. Moreover, mannose receptor CD206 is a typical marker for M2 macrophages, the augmentation of which usually represents a bad prognosis for cancer patients [27,28]. Studies showed that the mannosylation of HA coating particles effectively elevates the uptake by antigen-presenting cells, such as DCs and macrophages [26,29]. To explore whether our mannose-modified nanoparticles could actively target M2-like macrophages, IL-4-pretreated RAW264.7 cells were cultured with ferumoxytol, HA@Fe_3_O_4_, HA@Fe_3_O_4_-O_2_, and HA-man@Fe_3_O_4_. We first evaluated the cytotoxicity of the materials and then measured the intracellular iron content by Prussian blue spectrophotometry. CCK8 test results demonstrated that none of the materials exhibited significant cytotoxicity (Figure S2). Compared to the PBS groups, the IL-4 pretreated RAW264.7 cells displayed enhanced endocytosis of HA@Fe_3_O_4_ and HA-man@Fe_3_O_4_ (Figure 2C), thereby suggesting HA and mannose molecules could specifically enhance the targeting of M2-like macrophages. Indeed, HA coating molecules could also improve the targeting ability of macrophages by binding to CD44 on the cell surface [30]. The amount of HA@Fe_3_O_4_ in M2-like macrophages was approximately twice that of ferumoxytol following a coculture for 24 h (Figure 2A,B). HA-man@Fe_3_O_4_ further increased the targeting ability compared with HA@Fe_3_O_4_, owing to mannose modification (Figure 2A,B). HA@Fe_3_O_4_-O_2_ displayed a targeting ability similar to that of HA@Fe_3_O_4_ because of the identical surface molecules. The enhanced targeting ability of HA-man@Fe_3_O_4_ laid the foundation for the subsequent repolarization of macrophages.

### 3.3. Repolarization of M2-like Macrophages In Vitro

**Figure 3:**
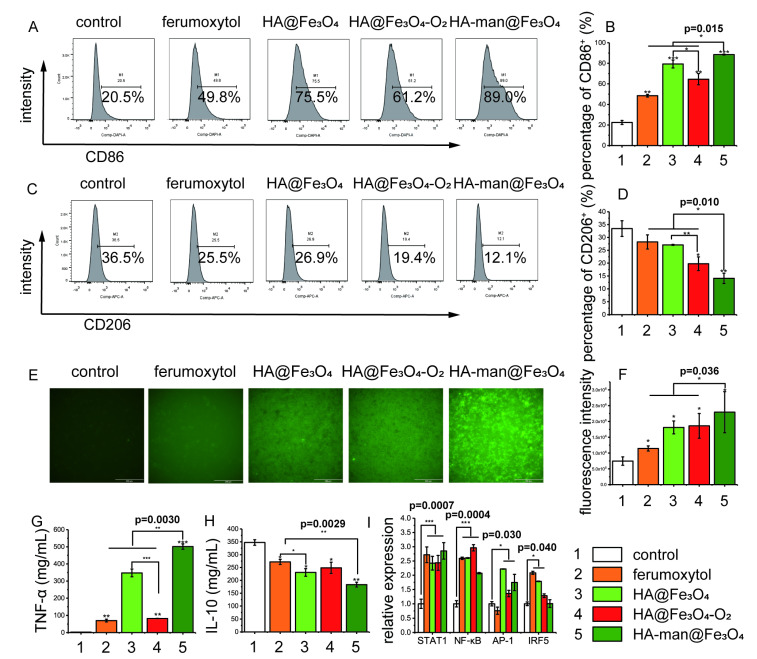
RAW 264.7 cell polarization in vitro. (**A**,**B**) Flow cytometry analysis of the expression of CD86 in RAW 264.7 cells; (**C**,**D**) flow cytometry analysis of the expression of CD206 in RAW 264.7 cells; (**E**,**F**) expression of intracellular ROS in RAW 264.7 cells; (**G**) TNF-α level in the cell culture media; (**H**) IL-10 level in the cell culture media; and (**I**) quantitative analysis of gene expression levels in RAW 264.7 cells. Cells were incubated with 200 μg mL^−1^ iron oxides in Fe for 24 h. Error bars indicate at least three independent experiments. Representative flow cytometry results are displayed. *p*-value has been calculated by the one-way ANOVA test. * *p* < 0.05, ** *p* < 0.01, and *** *p* < 0.001.

We sought to determine the effect on M2-like macrophage repolarization based on the enhanced targeting abilities. CD86 and CD206 are typical M1 and M2 markers, respectively. Therefore, we treated macrophages with various iron oxide nanoparticles and examined changes of the two markers after coincubation. The HA-man@Fe_3_O_4_-treated CD86^+^ cells expanded to approximately 90%, whereas the control group displayed only approximately 20% CD86^+^ cells (Figure 3A,B). Cells treated with commercialized ferumoxytol also promoted the expression of CD86 to approximately 50%, consistent with former studies. In addition, CD206 expression decreased significantly following treatment with HA-man@Fe_3_O_4_ (Figure 3C,D). Interestingly, the expression of CD86 and CD206 appeared correlated with the amount of endocytosis particles, suggesting that increased intracellular iron content could enhance the efficiency of repolarization.

Besides changes in surface makers, proinflammatory cytokines are also significant signals to reveal the state of macrophage repolarization. Therefore, we then collected the supernatant of culture medium for enzyme-linked immunosorbent assay (ELISA) after incubation with various nanoparticles. TNF-α is a classic inflammatory cytokine hypersecreted during the early stages of tumor growth, and is regarded as a signature product of M1 macrophages. In contrast, IL-10 is regarded as an important product of M2 macrophages, and plays an important role in tumor growth and development. Intriguingly, we found that TNF-α secretion significantly increased following stimulation with HA@Fe_3_O_4_ and HA-man@Fe_3_O_4_ for 24 h (Figure 3G). Its concentration in the HA-man@Fe_3_O_4_-treated culture medium was approximately five times higher than that in the ferumoxytol group. HA@Fe_3_O_4_-induced TNF-α secretion was blocked by oxidation, which might result from the reduction of ferrous ions in the particles during oxidation [31]. Moreover, HA-man@Fe_3_O_4_ inhibited IL-10 secretion (Figure 3H), thus indicating the repolarization of M2-like macrophages towards M1.

### 3.4. Effect of Macrophages on ROS Generation and Inflammatory Cell Pathways

Reactive oxygen species (ROS) are considered one of the mechanisms in iron oxide-mediated macrophage repolarization. Thus, we investigated the intracellular ROS and classic M1 signaling pathways to better understand the repolarization process. M1 macrophage polarization was closely related to four transcription factors, nuclear factor kappa B (NF-κB), signal transducer and activator of transcription 1 (STAT1), activator protein 1 (AP-1), and interferon regulator 5 (IRF5) [32]. After coincubation, we found that our dual-modified nanoparticles HA-man@Fe3O4 significantly increased the intracellular ROS levels of macrophages (Figure 3E,F) by threefold compared with the control group, whereas ferumoxytol increased the levels by 50% relative to the control group. This might explain the differences between our nanoparticles and ferumoxytol in macrophage repolarization, considering that ROS production could promote inflammation by polarizing the macrophages [33,34].

NF-κB expression levels are strongly associated with intracellular ROS levels. The expression was upregulated in all experimental groups, consistent with the ROS results (Figure 3I). The AP-1 expression level was elevated by HA@Fe_3_O_4_, HA@Fe_3_O_4_-O_2_, and HA-man@Fe_3_O_4_, but not ferumoxytol. This might be attributed to the immunogenicity of HA through the activation of TLR2 and TLR4. Interestingly, HA-man@Fe_3_O_4_ alleviated the iron-induced upregulation of IRF5, which might be caused by the degradation of transcription factors owing to prolonged stimulation.

### 3.5. Construction and Characterization of HA-man@Fe_3_O_4_@pep

**Figure 4:**
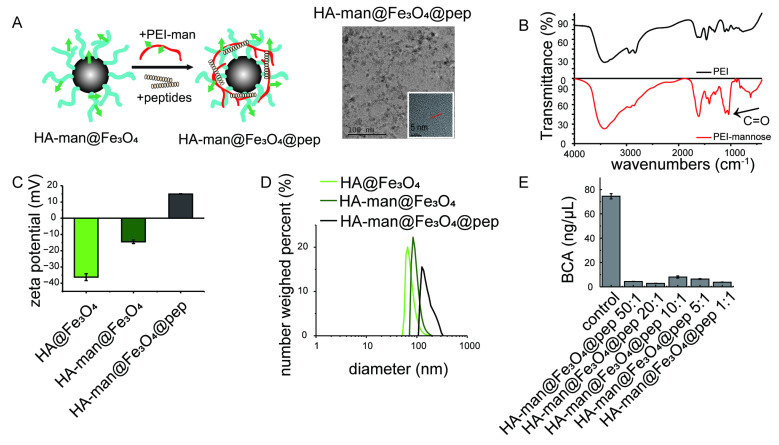
Characterization of HA-man@Fe3O4@pep nanocomplex. (**A**) Illustration of nanocomplex and TEM images of HA-man@Fe_3_O_4_@pep; (**B**,**C**) size distribution and zeta potentials of HA@Fe_3_O_4_, HA-man@Fe_3_O_4_, and HA-man@Fe_3_O_4_@pep; (**D**) infrared spectra of PEI and mannosylated PEI; (**E**) OVA concentration in the supernatant following adsorption. Error bars indicate at least three independent experiments.

Neoantigen-based cancer vaccines have drawn great attention in recent years. We assumed that neopeptides together with our dual-modified HA-man@Fe_3_O_4_ nanoparticles could enhance the efficacy because of its robust macrophage targeting and repolarization ability. We prepared the peptide-loaded nanoparticles (HA-man@Fe_3_O_4_@pep) by using commonly used cationic polymer, polyetherimide (PEI) to adsorb peptides. To ensure the quantity of mannose exposure, PEI was conjugated with mannose and the characteristic peak was observed (Figure 4B). The HA-man@Fe_3_O_4_@pep complex was packed together and the hydraulic diameter was increased to approximately 170 nm (Figure 4A,D). The zeta potential value changed to approximately + 15 mV following the construction (Figure 4C). This might result from numerous amino groups from PEI-man exposing in the outer layer, whereas the peptides were packed in the inner layer. The aforementioned type of structure thus might shelter peptides from complicated physiological environments and degradation. As this nanocomplex combines antigen peptides and HA-man@Fe_3_O_4_ adjuvant, we called HA-man@Fe_3_O_4_@pep nanovaccine. We then used a series of HA-man@Fe_3_O_4_ and ovalbumin (OVA) protein ratios to determine the loading efficiency from the remaining OVA in the supernatant. The loading capacity was maintained at approximately 85%, at whatever ratio, indicating that PEI-mannose still possessed cationic charges to adsorb negative molecules like protein or peptides (Figure 4E). This result was similar to that of Mooney’s group, who used PEI to build a complex, which confirmed that mannose modification did not hinder the adsorption ability of PEI [35]. Altogether, we constructed a nanovaccine based on our dual-modified nanoparticles with neopeptides and the efficacy was then explored. The peptides used in this study are detailed in Table 2.

**Table 2:** Sequence of peptides in article.

ID	Name	Sequence
1	HPVp1	ELQTTIHDI
2	HPVp2	LLMGTLGIV
3	HPVp3	YMLDLQPETT
4	HPVp4	QAEPDRAHYNIVTFCCKCD
5	OVA	SIINFEKL

### 3.6. Macrophages Mediated TC1 Inhibition In Vitro

**Figure 5:**
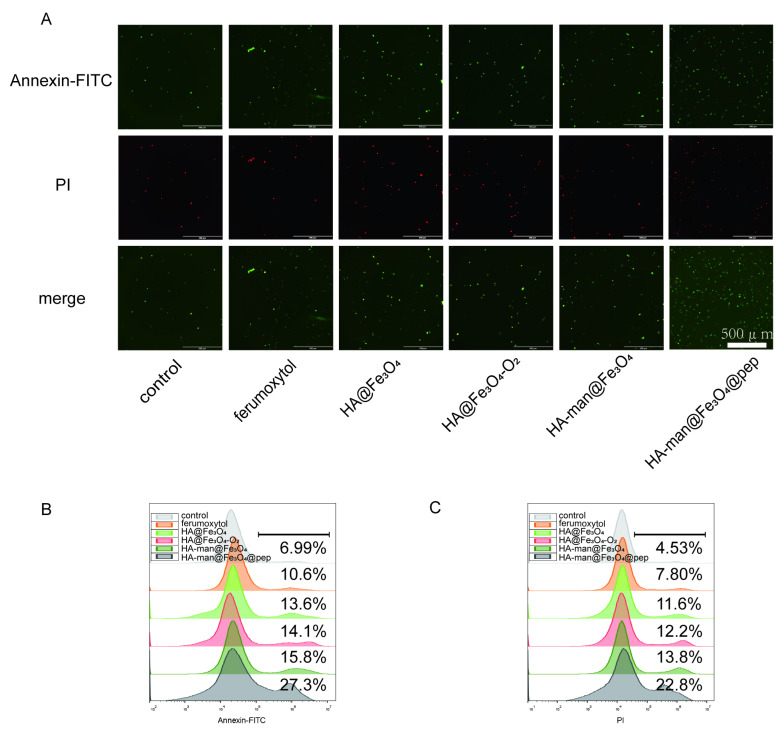
TC1 cell apoptosis in vitro mediated by RAW 264.7 and materials. (**A**) Fluorescence images of TC1 cells following coculturing with RAW 264.7 cells; (**B**,**C**) flow cytometry analysis of TC1 cells following coculturing with RAW 264.7 cells. Representative results are displayed.

To examine the effect of our nanovaccine, we used a TC1 cancer cell model, which is typical to study human papillomavirus related tumor with relatively clear epitopes derived from HPV16 E7 protein. To test the cytotoxicity of repolarized macrophages on cancer cells, TC1 cells were cocultured with RAW264.7 cells in a transwell system. RAW264.7 cells were simultaneously stimulated with ferumoxytol, HA@Fe_3_O_4_, HA@Fe_3_O_4_-O_2_, HA-man@Fe_3_O_4_, or HA-man@Fe_3_O_4_@pep separately before coincubation with TC1. As shown in Figure 5A–C, macrophage stimulation with HA-man@Fe_3_O_4_@pep significantly promoted the apoptosis of TC1 cells. HA-man@Fe_3_O_4_@pep-mediated apoptosis was approximately four times that of the control group, whereas ferumoxytol-mediated apoptosis was approximately twice that of the control group. HA@Fe_3_O_4_ and HA@Fe_3_O_4_-O_2_ demonstrated similar apoptosis levels, thus indicating that the oxidation did not affect the antitumor ability of macrophages. However, HA-man@Fe_3_O_4_ marginally increased the apoptosis rate compared with HA@Fe_3_O_4_, consistent with the ROS results. The positive rate of the late-stage apoptosis signal propidium iodide (PI) was approximately equal to the rate of the early apoptosis signal annexin-FITC, which indicated that the majority of TC1 cells entered the late stage of apoptosis within 24 h. Therefore, iron-induced macrophage-mediated apoptosis must be a rapid process.

### 3.7. Tumor Inhibition In Vivo

**Figure 6:**
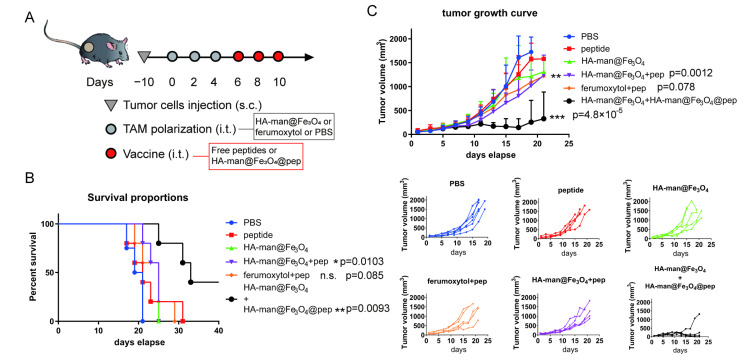
TC1 tumor inhabitation in vivo. (**A**) Scheme displaying the time schedule of the tumor implantation and treatment of C57 mice; (**B**) survival curves posttreatment; (**C**) average tumor growth curves and individual tumor growth curves posttreatment. Mice were treated with 20 mg kg^−1^ iron oxides in Fe and 2 mg kg^−1^ peptide per dose. Equal amount was achieved in HA-man@Fe_3_O_4_@pep. *p*-value has been calculated by the one-way ANOVA test. ** *p* < 0.01, and *** *p* < 0.001.

Immunosuppressive TME is one of the major hurdles for enhanced cancer vaccine therapy and TAMs are abundant and of significance to help tumor cells escape from immune supervision. Therefore, we hypothesized that HA-man@Fe_3_O_4_ might be used as adjuvant to first improve the immune condition “soil” in TME and then HA-man@Fe_3_O_4_@pep nanovaccine could promote efficacy further (Scheme 1). To test this strategy, we investigated the antitumor effect in a TC1 tumor-bearing mouse model in vivo and waited for tumor volume reaches 100 mm^3^ to start the therapy in order to mimic the real cases in cancer patients and ensure enough macrophage recruitment and infiltration. Only HA-man@Fe_3_O_4_ was tested because of its settled ability to polarize macrophages in vitro, and ferumoxytol was used in comparison because of its proven antitumor effect. First, HA-man@Fe_3_O_4_ and ferumoxytol were injected into the tumor to achieve TAM repolarization, followed by the use of peptides to induce the antitumor effect (Figure 6A). Compared with peptide vaccination alone, both HA-man@Fe_3_O_4_ and ferumoxytol increased the antitumor effect (Figure 6B). In addition, the inhibition of the HA-man@Fe_3_O_4_ + pep group was more effective than that of HA-man@Fe_3_O_4_, which demonstrated the effectiveness of the two-step vaccination. Moreover, the combination of HA-man@Fe_3_O_4_ and HA-man@Fe_3_O_4_@pep completely inhibited tumor growth for approximately 20 days, and two out of five mice in this group eventually achieved long-term survival (Figure 6B,C). Hence, the HA-man@Fe_3_O_4_ and peptide complex significantly improved the antitumor ability of the peptide vaccine.

### 3.8. TAM Repolarization and Enhanced T Cell Infiltration into Tumor

**Figure 7:**
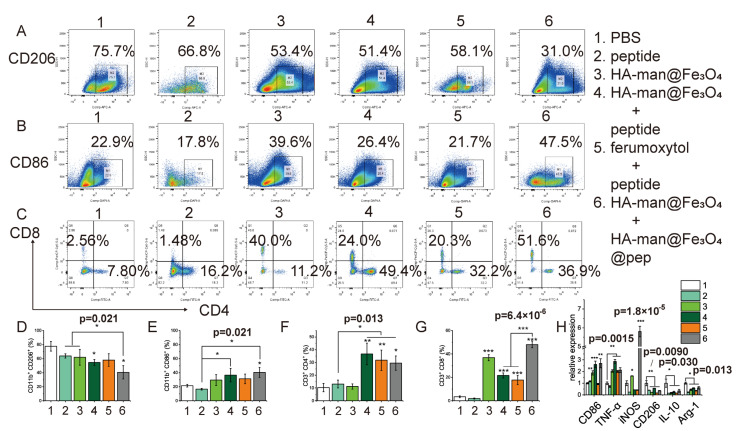
Flow cytometry and qPCR analysis of tumor tissue 24 h posttreatment. (**A**,**D**) CD206 expression by TAMs; (**B**,**E**) CD86 expression by TAMs; (**C**,**F**,**G**) CD4 and CD8 expression by T cells in tumor tissue; (**H**) TAM polarization-associated gene expressions of tumor tissue. Error bars indicate at least three independent experiments. *p*-value has been calculated by the one-way ANOVA test. * *p* < 0.05, ** *p* < 0.01, and *** *p* < 0.001.

To investigate TAM repolarization in the TME, tumor tissues from various treatment groups were collected and analyzed by flow cytometry. CD206 expression in macrophages decreased following HA-man@Fe_3_O_4_ treatment (Figure 7A,D). Combination therapy with HA-man@Fe_3_O_4_@pep further decreased its expression in the TME. Consistent with CD206 expression, CD86 expression in macrophages was elevated by HA-man@Fe_3_O_4_ and was further enhanced when combined with HA-man@Fe_3_O_4_@pep (Figure 7B,E). This enhanced polarization effect might be attributable to two reasons: (1) the iron particles further strengthened the polarization effect; and (2) the nanocomplex stimulated macrophages to be involved in the immune response. Compared with the peptide and HA-man@Fe_3_O_4_ groups, the HA-man@Fe_3_O_4_ + peptide group enhanced macrophage polarization. This might indicate that antigen stimulation plays an important role in macrophage polarization. We analyzed the total RNA of tumor tissue by RT-qPCR to confirm the repolarization effect. The expression of M1-related markers was remarkably upregulated by HA-man@Fe_3_O_4_, particularly in the HA-man@Fe_3_O_4_ + HA-man@Fe_3_O_4_@pep group (Figure 7H). Correspondingly, the expression of M2-related markers was downregulated in the HA-man@Fe_3_O_4_ and combination therapy groups. In summary, HA-man@Fe_3_O_4_ achieved M1-like macrophage polarization in vivo, and HA-man@Fe_3_O_4_@pep further enhanced the polarization effect.

Cytotoxic T lymphocytes are the central of immune cycles in cancer immunotherapy. Therefore, we then analyzed the infiltrated T cells to further investigate the effect of TAM repolarization on TME. The peptide group exerted a minute effect on CD4^+^ T cells in the tumor while CD4^+^ T cells significantly increased upon combining the peptides with iron particles (Figure 7C,F). HA-man@Fe_3_O_4_ alone failed to stimulate the promotion of CD4^+^ T cells, indicating the necessity of tumor-specific peptides in the infiltration of CD4^+^ T cells. In addition, the free peptides exerted a moderate influence on the CD8^+^ T cells (Figure 7C,G). Interestingly, HA-man@Fe_3_O_4_ significantly increased the infiltration of CD8^+^ T cells into the tumor tissue regardless of the presence of peptides (Figure 7C,G). HA-man@Fe_3_O_4_@pep further increased the infiltration of CD8^+^ T cells, compared with peptides alone. Moreover, CD8^+^ T cells in the ferumoxytol-combined peptide group also increased, in accordance with former report. In all, our dual-modified nanoadjuvant could repolarize TAMs towards M1 phenotype and improve the recruitment of CD8^+^ T cells into the tumor.

### 3.9. Enhanced Antigen Presentation by DCs in Sentinel Lymph Nodes

**Figure 8:**
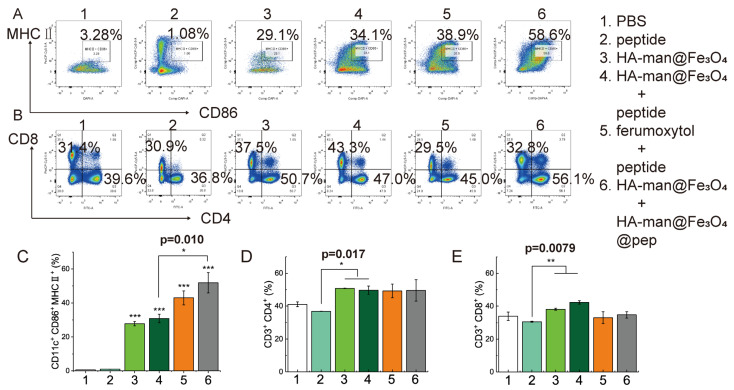
Flow cytometry analysis of sentinel lymph node tissue 24 h posttreatment. (**A**,**C**) Expression of maturation markers in DCs; (**B**,**D**,**E**) expression of CD8 and CD4 in T cells. Error bars indicate at least three independent experiments. *p*-value has been calculated by one-way ANOVA test. * *p* < 0.05, ** *p* < 0.01, and *** *p* < 0.001.

Dendritic cells are targeted in cancer vaccine immunotherapy and their maturation are of significance in immune cycles as potent APCs. Therefore, we analyzed the sentinel lymph nodes for antigen presentation 24 h posttreatment. CD86 and MHC II markers on DCs were analyzed by flow cytometry as signals of DC maturation. The HA-man@Fe_3_O_4_ group remarkably promoted the maturation of DCs compared with the control group, whereas the free peptide group exerted little influence (Figure 8A,C). The HA-man@Fe_3_O_4_ combined with HA-man@Fe3O4@pep group exerted more significant influence on DC maturation compared with the HA-man@Fe_3_O_4_ or HA-man@Fe_3_O_4_ combined peptide groups. Interestingly, the ferumoxytol combined peptide group also promoted DC maturation, thus indicating iron ions may be an essential element of DC maturation.

T cells in the sentinel lymph nodes were also analyzed. All experimental groups exerted little influence on CD4^+^ T cell infiltration, compared with the control group (Figure 8D). Correspondingly, the experimental groups did not exert significant influence on CD8^+^ T cells in the sentinel lymph nodes, except the HA-man@Fe_3_O_4_ + peptide group (Figure 8E). In all, our dual-modified iron oxide, HA-man@Fe_3_O_4_ exert a robust effect on TAM repolarization in TME, modulating DC maturation both in TME and lymph nodes and promoting CD8^+^ T cell infiltration in TME.

## 4. Discussion

Neoantigen-based cancer vaccination therapy has drawn much attention during the past decade due to its personalized characteristic and its minute side effect [36]. Early clinical trials have proved the efficacy of neoantigen vaccine, which usually induce memory T cell responses and tumor infiltration of neoantigen-specific T cell clones [1,2]. However, the tumor microenvironment impedes T cell response with a stiff matrix to prevent T cell entering and immunosuppressive cells in TME like TAMs further support tumor angiogenesis. Therefore, repolarizing TAMs is a rational strategy to converting “cold tumors” to “hot tumors” [37,38].

Emerging nanoparticles have been developed to target TAMs for enhancing immunotherapy, including lipid-based nanomaterials, polymers and inorganic nanomedicine [39]. Among them, iron oxide nanoparticles might be antitumor arsenals. They have been used in clinics as MRI contrast agent for decades and have recently gained interest for their ability to orchestrate macrophage polarization [40]. An RNA vaccine designed for COVID-19 also used iron oxide to enhance vaccine stability and immunogenicity [41,42], indicating that iron oxide-based nanovaccines might combine adjuvant effects with carrier function and could enter clinical application in the future if their efficacy and safety are of satisfaction.

In this study, we prepared a hyaluronic acid and mannose dual-modified iron oxide nanoadjuvant, HA-man@Fe_3_O_4_. The coating and modification were confirmed using zeta potential and infrared spectroscopy (Figure 1). HA and iron oxide were used to induce TAMs to shift from suppressive to an acute inflammatory phenotype, while mannose was leveraged to bind to the mannose receptor, which is usually expressed in TAMs. It takes advantage of the plasticity of macrophages and its natural location in tumor mass. As shown in Figure 2, more than 70% macrophages phagocytized this nanoadjuvant and only around 30% macrophages endocytosed FDA-approved agent ferumoxytol, indicating our modification contributed to enhanced macrophage targeting. Interestingly, IL-4-induced M2-like macrophages phagocytosed more nanoparticles compared to PBS-treated M0 macrophages in both HA modified iron oxide nanoparticles and dual-modified HA-man@Fe_3_O_4_ (Figure 2C), which further satisfied the need of repolarizing TAMs that have been educated by cancer cells for a long time and turned M2-like. Of note, dextran-decorated iron oxide nanoparticle ferumoxytol has been reported to have antitumor [43] and M2 repolarization effects [17], which were further confirmed in our study (Figure 3A–D, Figure 3G,H). Ferumoxytol did enhance CD86 expression and TNF-α production and downregulated CD206 with decreased IL-10, while our nanoparticles HA-man@Fe_3_O_4_ outperformed it in macrophage repolarizing with the same amount of iron, suggesting our nanoadjuvant might be efficient in cancer vaccine treatment with HA and mannose dual modification. ROS is considered a mechanism for iron-related macrophage repolarization and we also tested ROS release after incubating macrophages with various iron oxide nanoparticles (Figure 3E,F). Our dual-modified nanoadjuvant induced the most ROS in macrophages, which probably contributed to the enhanced intake and different signal pathways triggered by various nanoparticles.

Based on our findings, we then hypothesized that this dual-modified nanoadjuvant could help improve the efficacy of neoantigen-based vaccines by first reversing TME from “cold” to “hot.” Interestingly, HA-man@Fe_3_O_4_ also empowered tumor apoptosis to double the extent of the control (Figure 5). With neopeptides, HA-man@Fe_3_O_4_@pep could further enhance TC1 tumor apoptosis at four times that of control in the presence of macrophages in transwell system, suggesting skewed M1 macrophages could secrete cytotoxic matters to kill tumor cells.

Similar to our design, a study combined a polymeric nanoadjuvant with peptide-based vaccines to repolarize TAMs and meanwhile induce cytotoxic T cells [44]. The polymeric nanocarrier was loaded with CCL20, poly (I:C) and R848, among which poly (I:C) and R848 own repolarization effects on macrophages [44]. The study proved that the combination of converting TAMs and cancer vaccine prompted the efficiency of vaccination, which is in line with our observation in this study. Instead of injecting peptides and immune modulators separately, our nanoadjuvant could directly load with tumor-specific peptides as carrier and the inhibition effect in TC1 model was comparable as 40% of mice underwent complete regression (Figure 6). An iron oxide nanoparticle coated with HA was reported recently and it could repolarize macrophages and suppressed 4T1 tumor growth with or without magnet [45]. This HA-coated nanoparticles could increase CD86^+^ macrophages and stimulate macrophages to secrete more TNF-α, which is in accordance with our study (Figure 3A,B,G).

Moreover, intratumoral administration of HA-man@Fe_3_O_4_-based nanovaccine profoundly impacted immune cells in the tumor and tumor-draining lymph node. In tumor tissue, the presence of CD206^+^ macrophages (M2-like) dropped from nearly 80% to 40%, while CD86^+^ macrophages rose from around 20% to 40% (Figure 7). As for CD8^+^ T cell infiltration, this HA-coated nanoparticle promoted it from 3.78% to 20.3% [45], while our nanoparticles induced nearly 12-fold compared to PBS control and further increased to about 17-fold in combination with neopeptide-loaded nanovaccine (Figure 7). In sentinel lymph nodes, mature DCs (identified as CD86^+^MHC II^+^) increased robustly in the HA-man@Fe_3_O_4_ plus HA-man@Fe_3_O_4_@pep group, while free peptides failed to arouse mature DCs, suggesting the necessity of adjuvant and carrier in cancer vaccination (Figure 8). Indeed, ferumoxytol administration following peptides could also repolarize TAMs, only at a relatively weak level, which might explain the unsatisfactory efficacy in tumor growth suppression. We also performed q-PCR to examine the expression of mRNA-encoded M1 and M2 markers and discovered that M1-associated CD86, TNF-alpha and iNOS were all improved in the HA-man@Fe_3_O_4_ plus HA-man@Fe_3_O_4_@pep group, while M2 markers were decreased (Figure 7H). Notably, iNOS in group 6 was drastically promoted six-fold compared to PBS control, which might contribute to the potent efficacy of this combination therapy. The HA-man@Fe_3_O_4_ with free peptide group (group 4) also improved the level of CD86 and TNF-alpha. In contrast, ferumoxytol plus peptide could only affect TNF-alpha expression and failed to induce iNOS and CD86 (Figure 7H), indicating its unsatisfactory converting effect on TME. In all, these results suggested that our nanoparticles could not only target and repolarize TAMs but propagate immune responses in vivo.

Furthermore, we sought to broaden our understanding of the mechanisms of iron oxide-based macrophage repolarization and tumor inhibition. Fenton reaction has been considered as the innate reason for macrophage conversion and ROS is one significant production mediated the process [16,46]. Thus, we examined the ROS releasing from macrophages and found that HA-man@Fe_3_O_4_ induced the highest ROS amount in all iron oxide-treated groups, corresponding to the strongest skewing effect shown in flow cytometry and ELISA assay (Figure 3A–H). Two commercialized iron oxide nanoparticles including ferumoxytol have been reported to activate TLR4 signal pathway of macrophages and promote the expression of proinflammatory cytokines [47]. Therefore, we also explored the expression of molecules in TLR-related signal pathway in iron oxide-stimulated macrophages (Figure 3I). Intriguingly, iron oxide nanoparticles appeared to induce several transcription factors, including STAT1, NF-kB and AP-1. The gene expression profile of HA-man@Fe_3_O_4_-induced macrophages was different from ferumoxytol and HA@Fe_3_O_4_ and this phenomenon warrants further exploration in future study.

The physicochemical properties of nanoparticles determine whether they are a satisfactory adjuvant. As aforementioned, iron-containing nanoparticles are promising candidates because of their potent repolarization effect on macrophages and their natural existence and metabolism in the human body. Reports have suggested that antigen-presenting cells (APCs) including DCs and macrophages prefer phagocytosing larger particles [48], which might contradict with iron-based contrast agents that require smaller size to drain lymph nodes and substantial mass. With HA coating and mannose decoration, the iron oxide nanoparticles could reach nearly 80 nm and the cluster formed with PEI-man and peptides further reached 170 nm, resulting from decreased surface potential (Figure 1B and Figure 4D). This enhanced size might promote APC endocytosis. Of note, the safety profile of our nanoadjuvant HA-man@ Fe_3_O_4_ remained comparable to that of ferumoxytol (Figure S2). In addition, the cationic surface charges of HA-man@Fe_3_O_4_@pep might also enhance the availability of cells because of the negative charges of cellular membrane and improve delivery cargos to target cells. Indeed, this nanoadjuvant HA-man@Fe_3_O_4_ could also be versatile to be used in combination with other antigenic molecules, such as plasmid DNA, message RNA and antigen proteins. Although the tumor microenvironment is complicated and this nanoadjuvant requires further preclinical and clinical studies, it still provide references for the future development of rationale design nanoparticles and the selection of adjuvants.

## 5. Conclusions

In summary, we developed HA-man@Fe_3_O_4_, a hyaluronic acid and mannose co-modified Fe_3_O_4_ nanoparticle, to efficiently target and repolarize TAMs in the TME. HA-man@Fe_3_O_4_ could switch the M2-like TAM into proinflammatory M1-like macrophages both in vitro and in vivo and our nanoadjuvant-polarized macrophages were able to induce TC1 cancer cell apoptosis. We hypothesized that this nanoadjuvant could first improve the “soil” in TME because of TAM repolarization and then enhance the efficacy of neopeptide-based cancer vaccine. Indeed, combined therapy with neoantigen-based peptide vaccines significantly inhibited tumor growth and enhanced the antitumor effects in TC1 model to reach 40% complete regression. HA-man@Fe_3_O_4_ enhanced T cell infiltration in tumors and significantly promoted DC maturation in sentinel lymph nodes. In summary, HA-man@Fe_3_O_4_ has been proven to be a low-cytotoxicity and highly efficient adjuvant for targeting and repolarizing macrophages. Combination therapy based on HA-man@Fe_3_O_4_ iron particles and its nanocomplex system provides a generalizable method to enhance the effectiveness of neoantigen-based peptide cancer vaccines.

## Data Availability

All data described in this article are available from the corresponding author on reasonable request.

## Supplementary Materials

The following supporting information can be downloaded at: https://www.mdpi.com/article/10.3390/cancers14205107/s1. Figure S1: The XRD spectrum of HA@Fe_3_O_4_ and HA@Fe_3_O_4_-O_2_; Figure S2: The CCK8 results of HA@Fe_3_O_4_, HA@Fe_3_O_4_-O_2_, HA-man@Fe_3_O_4_ and ferumoxytol; Table S1: Sequence of qPCR primers in article_._
