# Supplementary material for: Mannose and Hyaluronic Acid Dual-Modified Iron Oxide Enhances Neoantigen-Based Peptide Vaccine Therapy by Polarizing Tumor-Associated Macrophages
Source: Cancers (Basel). 2022 Oct 18;14(20):5107. doi: 10.3390/cancers14205107 (PMC9599981; doi:10.3390/cancers14205107)
Supplement: Supplementary file 1 [file cancers-14-05107-s001.zip › cancers-1939802-supplementary.pdf]

Mannose and Hyaluronic Acid Dual-modified Iron Oxide En-hances Neoantigen-based Peptide Vaccine Therapy by Polar-izing Tumor-associated Macrophages

Ying Nie [1,†], *Lu Shi* [1,†], *Yanan Zhang* [1], *Yunfei Guo* [1] *and Hongchen Gu* [1,*]

Supporting Information

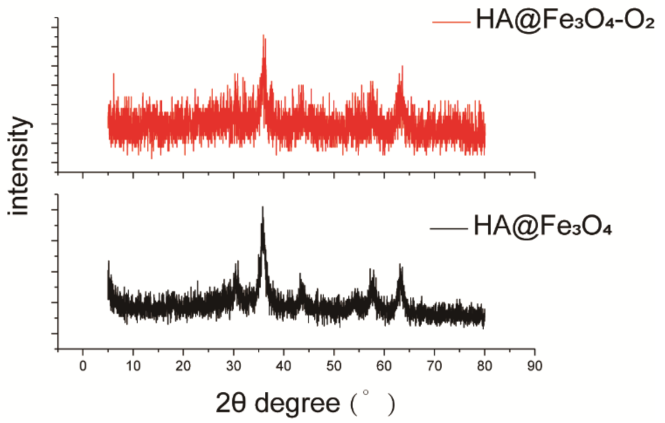

Supplementary Figure S1. The XRD spectrum of HA@Fe_3O_4 and HA@Fe_3O_4-O_2.

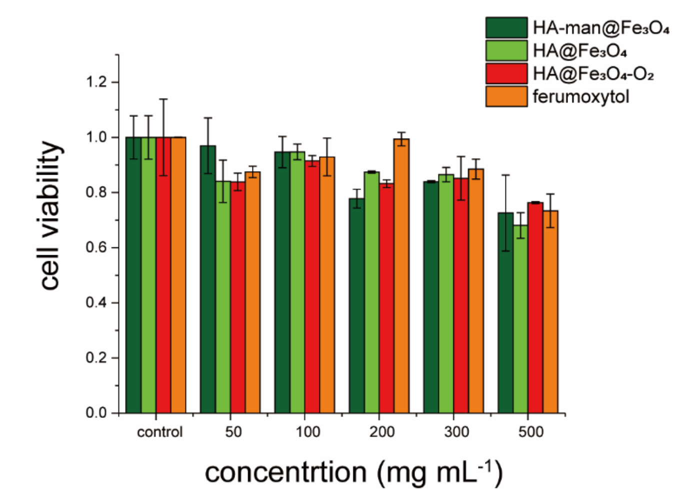

Supplementary Figure S2. The CCK8 results of HA@Fe_3O_4, HA@Fe_3O_4-O_2, HA-man@Fe_3O_4 and ferumoxytol. Raw264.7 cells were co-cultured with materials for 24 hours before test.

Supplementary Table S1. Sequence of qPCR primers in article.

Name	Forward	Backward
STAT1	5′-GTCATCCCGCAGAGAGAACG-3′	5′-GCAGAGCTGAAACGACCTAGA-3′
NF- κB	5′-CCTGCTTCTGAGGGTGATG-3′	5′-GCCGCTATATGCAGAGGTGT-3′
AP-1	5′-TTGTTACAGAAGCGGGGACG-3′	5′-GAGGGCATCGTCGTAGAAGG-3′
IRF5	5′-CCCTGTCCCAGACCCAAATC-3′	5′-AGGTCCGTCAAAGGCAACAT-3′
iNOS	5′- GTTCTCAGCCCAACAATACAA GA-3′	5′- GTGGACGGGTCGATGTCAC-3′
CD86	5′- TCAATGGGACTGCATATCTGCC-3′	5′- GCCAAAATACTACAGCTCACT-3′
Arginase I	5′- TGTCCCTAATGACAGCTCCTT-3′	5′- GCATCCACCCAAATGACACAT-3′
CD206	5′- AGGCTGATTACGAGCAGTGG-3′	5′- CCATCACTCCAGGTGAACCC-3′
β-actin	5′- GGAGATTACTGCCCTGGCTCC TA-3′	5′-GACTCATCGTACTCCTGCTTGC TG-3′
IL-10	5′-GCATGGCCCAGAAATCAAGG-3′	5′-GAGAAATCGATGACAGCGCC-3′
TNF-α	5′- CCATCACTCCAGGTGAACCC-3′	5′- CGATCACCCCGAAGTTCAGTAG-3′
